# Translation, Cross-Cultural Adaptation, and Psychometric Testing of Yoruba Version of the EQ-5D Questionnaire in Patients With Musculoskeletal Disorders

**DOI:** 10.3389/fpubh.2022.902680

**Published:** 2022-06-27

**Authors:** Francis Fatoye, Abiodun Emmanuel Akinfala, Tadesse Gebrye, Clara Fatoye, Titilope Faith Ojelade, Olufemi Oyeleye Oyewole, Chidozie Emmanuel Mbada

**Affiliations:** ^1^Department of Health Professions, Manchester Metropolitan University, Manchester, United Kingdom; ^2^Department of Medical Rehabilitation, College of Health Sciences, Obafemi Awolowo University, Ile-Ife, Nigeria; ^3^Department of Health and Social Care, University Campus Oldham, Manchester, United Kingdom; ^4^Department of Physiotherapy, Olabisi Onabanjo University Teaching Hospital, Sagamu, Nigeria

**Keywords:** cross-cultural adaptation, EQ-5D questionnaire, Yoruba, musculoskeletal disorders, psychometric properties, translation

## Abstract

**Background:**

The EuroQol-5 Dimension (EQ-5D) is a generic self-administered questionnaire used for the measurement and economic valuation of a wide range of health conditions, which necessitates its existence and adaptation in different languages. Currently, the tool does not exist in any Nigerian language. This study aimed to translate, cross-culturally adapt, and determine the reliability and validity of the Yoruba version of the EQ-5D-5L questionnaire.

**Methods:**

The International Quality of Life Assessment (IQOLA) project guidelines, involving forward translation, reconciliation and harmonization, backward translation, and reconciliation of problematic items were used in the Yoruba translated version of the EQ-5D-5L (EQ-5D-Yor). A total of 113 and 109 persons with musculoskeletal disorders participated in the validity and 7-day test-retest reliability testing of the EQ-5D-Yor. Convergent and discriminant validity of the EQ-5D-Yor were determined using the Yoruba version of the 12-Item Short-Form Health Survey (SF-12) (SF-12-Y) and Visual Analog Scale (VAS). Data were analyzed using descriptive and inferential statistics of Spearman correlation, Intra-Class Correlation, Cronbach alpha, and multi-trait scaling analysis. Alpha level was set as *p* < 0.05.

**Results:**

The construct validity of the EQ-5D-Yor yielded Spearman rho ranging from 0.438 to 1.000, with the EQ-VAS having the highest co-efficient (*r* = 1.000; *p* = 0.001). The convergent validity of the EQ-5D-Yor index with scales and domains of the SF-12-Y yielded no significant correlations (*p* < 0.05), except for the physical functioning scale (*r* = −0.709, *p* = 0.001). On the other hand, the divergent validity of the EQ-5D-Yor index with VAS yielded a moderate negative correlation (*r* = −0.482; *p* = 0.001). The Intra-class Correlation Coefficient (ICC) and Cronbach's alpha for the test-retest reliability of the EQ-5D-Yor were 1.000 and 0.968. The confirmatory factor analysis showed the factor loadings were poor when including VAS in the model.

**Conclusion:**

The EQ-5D-Yor has acceptable validity and reliability and can be used as a valid tool among Yoruba speaking population with musculoskeletal disorders.

## Introduction

The need to measure the impact of disease conditions and the outcomes of intervention from the patient's point of view has necessitated the measurement and valuation of health-related quality of life (HRQoL). In turn, HRQoL data are important to generate Quality Adjusted Life Years (QALYs), which is a health utility metric of the value and benefit of health outcomes that is typically used as a basis to assess the clinical and cost-effectiveness of interventions during a clinical trial and in routine care setting (1). To assess HRQoL, a plethora of questionnaires have been developed and are frequently translated for use in languages and cultures other than the original. Some of these questionnaires include the Health Utilities Index 2 and 3 (HUI2 and HUI3), the Short Form 6 Dimension (SF-6D), EQ-5D, and SF-12 (2). The SF-6D (physical activities, role limitations, social functioning, pain, mental health, and vitality) is one of the multi-attribute utility instruments derived from the SF-12, which consists of 12 items: general health, role limitations (2 items), physical health (2 items), emotional problems (2 items), pain, mental health (3 items), and social activities (2).

Among the various questionnaires, the EQ-5D has become the most ubiquitous generic too for measurement and economic valuation of a wide range of health conditions (3). The EQ-5D was developed by EuroQol Group in 1990 as an instrument which included a “common core” of content related to self-perceived health status. It was designed to “capture the key descriptive elements in which everybody was likely to be interested, not to create an exhaustive system for descriptors which would serve all purposes” (4).

Given the worldwide use of the EQ-5D as a patient-reported outcome tool for the measurement and valuation of health, researchers have often translated the EQ-5D questionnaire into their own languages (often with assistance from professional translators). The cross-cultural diversity of the population worldwide necessitates translations and adaptations of tools into different languages. Consequently, clinicians and researchers require psychometrically sound tools that measure the concepts/constructs of interest in their own cultures and languages. The availability of these tools in other languages promotes cross-cultural research which makes it possible to compare or aggregate results obtained in different countries. Sometimes, it also provides a measure in a given cultural setting where none existed before, which is important to ensure that equivalence between translated versions is maintained (5).

Currently, several translations of the EQ-5D exist in languages such as Korean (6), Singaporean Malay and Tamil (7), and Spanish (8). The only translation of the EQ-5D in sub-Saharan Africa is the Chichewa in Malawi (9). Nigeria represents the largest black population in sub-Saharan Africa, and the Yoruba language is one of the major native ethnic languages in Nigeria and other West African countries such as the Benin Republic, and parts of Togo and Sierra Leone. The objective of this study was to cross-culturally adapt and determine the psychometric properties of the Yoruba version of the EQ-5D (EQ-5D-Yor).

## Materials and Methods

Respondents for this study were community-dwelling residents of Ile-Ife, Osun State, Nigeria with a history of musculoskeletal disorders, who were 18 years and older and were literate in both English and Yoruba languages. Those excluded from the study were those with self-report history of psychiatric history or any systemic illness such as a tumor.

The sample size for this study, based on our calculations, was 103 respondents. To accommodate for non-completion or invalid data, 10% of the sample size was added, thus resulting in 113 respondents. Data collection continued until sample size was satisfied.

### Instrument

#### EQ-5D

The EQ-5D version that was used in this study was EQ-5D-5L. The descriptive system of EQ-5D-5L is a five-dimensional questionnaire with the following domains: mobility, self-care, usual activities, pain/discomfort, and anxiety/depression (10). Each question in the EuroQol-5 Dimensional questionnaire has 5 levels. The levels are rated as “no problems,” “slight problems,” “moderate problems,” “severe problems,” and “extreme problems”. As a self-administered tool, the respondent indicates his/her health state by checking the box that corresponds to the most appropriate statement in each of the five dimensions. The five dimensions have a 5-digit number that is used to describe the health state of the respondent. The EQ-5D-5L also has a visual analog scale (EQ-VAS) which helps to assess the self-perceived health of an individual as a single overall rating on a scale of 0–100 mm signifying “the worst” and “the best” health imaginable, respectively. The psychometric properties of the EQ-5D have been reported across a wide range of contexts and conditions to be excellent (11).

#### The Visual Analog Scale

The Visual Analog Scale (VAS) is a generic assessment tool that is often employed to measure a phenomenon that easily cannot be measured directly but is believed to range across a continuum of values (12). The VAS is a psychometrically sound instrument that is typically employed in the assessment of pain but has been adopted to measure disease-related symptoms and other constructs. The Yoruba version of the VAS that was employed in this study was reported to be usable and reliable among Yoruba-speaking populations (13).

#### The 12-Item Short-Form Health Survey

The SF-12 is an abridged version of the SF-36, which was originally developed for the Medical Outcomes Study, a multi-year study of patients with chronic conditions (14). Similar to the SF-36, the SF-12, as a shorter variant, uses the same eight scales and two domains. These scales are physical functioning (PF), role limitations due to physical problems (RLP), bodily pain (BP), role limitations due to mental health (RLM), social functioning (SF), and mental health (MH). These scales are then pooled together to form the Mental Health Domain (MHD) and the Physical Health Domain (PHD) (14).

The Yoruba version of the SF-12 has excellent psychometric properties with an internal consistency score range of 0.899 and 0.968 (Cronbach's Alpha), and an Intraclass Correlation Coefficient (ICC) range between 0.775 and 0.949. It has been employed in some earlier studies owing to its psychometrics and the language preference among Yoruba-speaking populations (15, 16).

### Procedure

The English version of the EQ-5D-5L was translated to the Yoruba language based on the stepwise EuroQol Version Management Committee (EQ-VMC) guidelines (17). The methodological process included two forward translations.

i. Two forward translations were carried out independently. Two qualified and experienced translators who were native Yoruba speakers and also fluent in the English Language performed this task.
ii. A first reconciled version of the Yoruba version of the EQ-5D questionnaire was produced by a local investigator with inputs from the forward translators by comparing and merging the two forward translations into a single forward translation.
iii. Thereafter, the single forward translation was reviewed by a reviewer.
iv. After the review, the single forward translation was back-translated. Two back translations were performed independently by qualified and experienced translators who were fluent in both English and Yoruba languages (See Appendix for the EQ-5D-Yor).

Ethical approval for the study was obtained from the Health Research Ethics Committee, Institute of Public Health, College of Health Sciences, Obafemi Awolowo University, Ile-Ife, Nigeria (IPH/OAU/12/1803).

### Data Analysis

Data were summarized using descriptive statistics of mean, standard deviation, and percentages. Inferential statistics of Spearman and Intra-Class Correlation analyses were used to confirm the relationship between the English and Yoruba version of the EQ-5D-5L. A scattered plot was used to depict the correlation between variables. Multi-trait scaling analysis was used to confirm the item's convergent [this denotes how closely the new tool (EQ-5D-Yor) is correlated to other measures of the same construct (Yoruba version of the SF-12)] and divergent (this refers to how distinctive the new tool is when compared with another similar tool that measures different constructs or traits) validity. Confirmatory factor analysis (CFA) and structural equation modeling were used to test the relationship of the tools with each other. Data were analyzed using SPSS (Statistical Package for Social Sciences) version 16.0 with an alpha level of *p* < 0.05.

## Results

The mean age of the respondents was 33.2 ± 13.31 years. The socio-demographic characteristics of the respondents are presented in Table 1. The mean score, standard deviation, kurtosis, and skewness of the Yoruba version of the EQ-5D questionnaire are presented in Table 2. The average mean scores for the items ranged from 1.21 to 73.54. Item 6 had the highest mean (73.54), while item 2 had the lowest mean (1.21).

**Table 1:** Socio-demographic descriptive characteristics of the respondents.

**Variable**	**Frequency**	**Percentage**
**Age group**		
<20 years	19	16.8
21–30 years	37	32.7
31–40 years	24	21.2
41–50 years	15	13.3
51–60 years	18	15.9
**Sex**		
Male	62	54.9
Female	51	45.1
**Educational level**		
Low	73	64.6
Medium	35	31.0
High	5	4.4

**Table 2:** Descriptive summary of each of the items of the EQ-5D-Yor (Mean score, standard deviation, Skewness, and kurtosis).

**Item**	**Mean**	**Median**	**SD**	**Kurtosis**	**Skewness**
1	1.33	1.00	0.47	−1.47	0.745
2	1.21	1.00	0.41	0.032	1.425
3	1.26	1.00	0.43	−0.738	1.129
4	1.26	1.00	0.46	0.719	1.409
5	1.31	1.00	0.46	−1.328	0.834
EQ-5D-Yor (VAS)	73.54	70.00	18.91	−1.183	0.077
EQ-5D-Yor (Sum)	6.36	6.00	1.30	−0.763	0.553

*SD, Standard Deviation; 1-5, EQ-5D-Y Scale items; EQ-5D-Yor (VAS), Yoruba translated EuroQol Visual Analog Scale; EQ-5D-Yor (Sum), Sum of all items of the Yoruba translated EuroQol*.

### Concurrent Validity of the EQ-5D-Yor

Table 3 shows the results of the concurrent validity of the EQ-5D-Yor by correlating the English and Yoruba versions of the EQ-5D. The Spearman correlation (*r*) of items of the EQ-5D-Yor were within the range of 0.438 and 1.000. The highest and the lowest scores were for item 6 (*r* = 1.000, *p* = 0.001) and item 3 (*r* = 0.438, *p* = 0.001).

**Table 3:** Concurrent validity, Cronbach's alpha, internal consistency of the EQ-5D-Yor questionnaire.

**Item**	**Concurrent validity**		**EQ-5D-Yor with EQ-5D English version**			**EQ-5D-Yor test-retest**		
	***r***	***p*** **-value**						
			**Cronbach α**	**ICC (95%CI)**	***p*** **-value**	**Cronbach α**	**ICC (95%CI)**	***p*** **-value**
1	0.604	0.001	0.751	0.751 (0.638–0.828)	0.001	0.972	0.972 (0.959–0.981)	0.001
2	0.553	0.001	0.711	0.711 (0.581–0.801)	0.001	0.937	0.937 (0.908–0.957)	0.001
3	0.438	0.001	0.608	0.608 (0.432–0.730)	0.001	0.965	0.965 (0.949–0.976)	0.001
4	0.546	0.001	0.678	0.678 (0.532–0.778)	0.001	0.977	0.977 (0.966–0.984)	0.001
5	0.669	0.001	0.802	0.802 (0.712–0.863)	0.001	0.928	0.956 (0.899–0.981)	0.001
EQ-5D-Yor (VAS)	1.000	0.001	0.853	0.853 (0.787–0.899)	0.001	0.962	0.962 (0.945–0.974)	0.001
EQ-5D-Yor (Sum)	0.733	0.001	1.000	1.000 (1.000–1.000)	0.001	0.968	0.968 (0.954–0.978)	0.001

*EQ-5D-Yor scale, Yoruba translated EuroQol scale; EQ-5D-Yor (VAS), Yoruba translated EuroQol Visual Analog Scale; EQ-5D-Yor (Sum), Sum of all items of the Yoruba translated EuroQol*.

### Reliability of the EQ-5D-Yor

**Figure 1:**
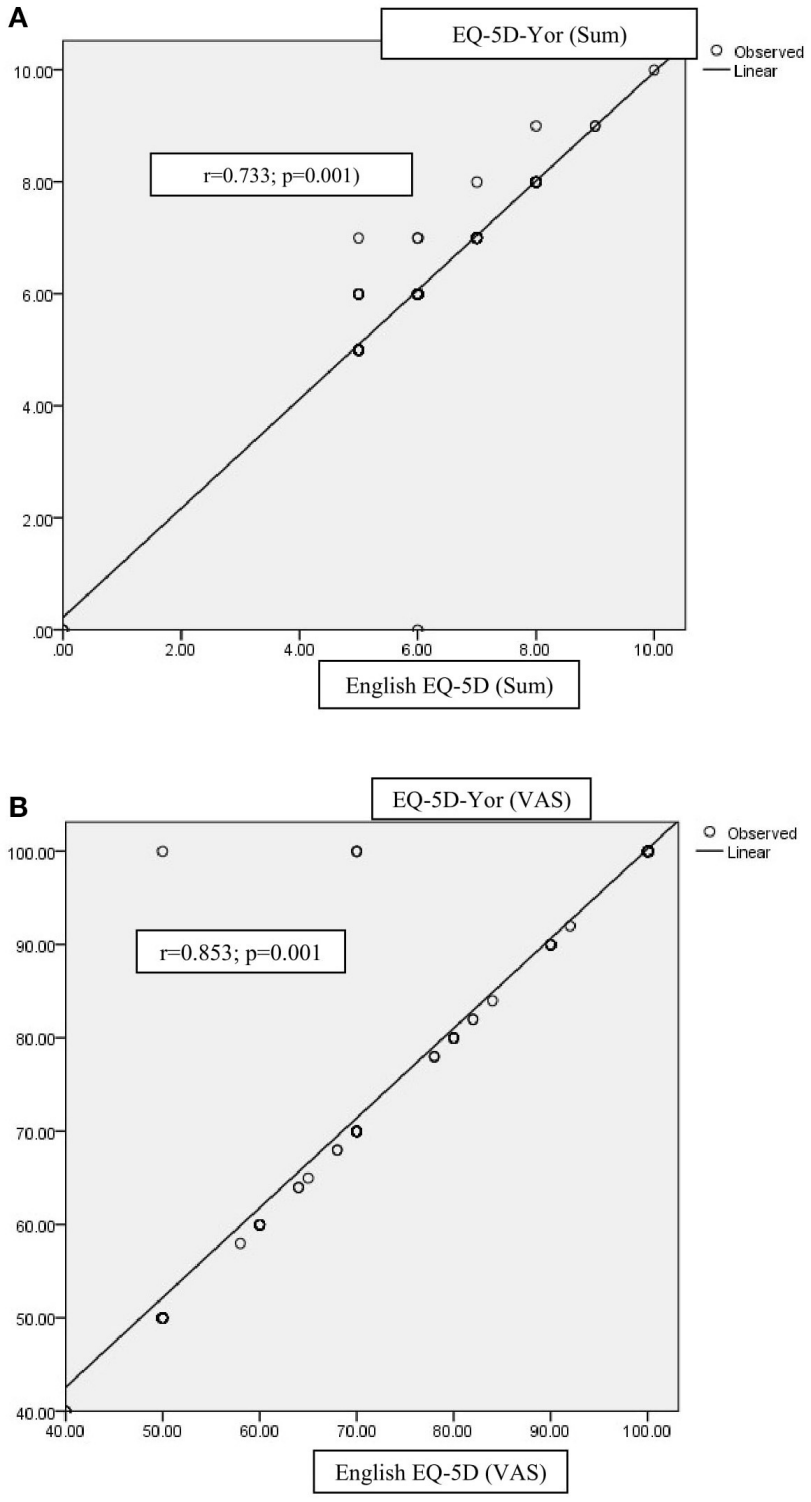
**(A)** Scattered plot diagram depicting the concurrent validity of the EQ-5D-Yor. **(B)** Scattered plot diagram depicting the correlation between the EQ-5D-Yor (VAS) and the English EQ-5D-(VAS).

The test-retest reliability of the EQ-5D-Yor within 7-days interval results yielded acceptable item scores ranging between 0.928 and 0.977 (Table 3). The test-retest reliability of the EQ-5D-Yor total score based on ICC yielded a perfect score of 1.00 [95% CI (1.00–1.00)]. Figure 1 is a scattered plot diagram that depicts the correlation between the test-retest of the EQ-5D-Yor.

### Common Factor Analysis

**Figure 2:**
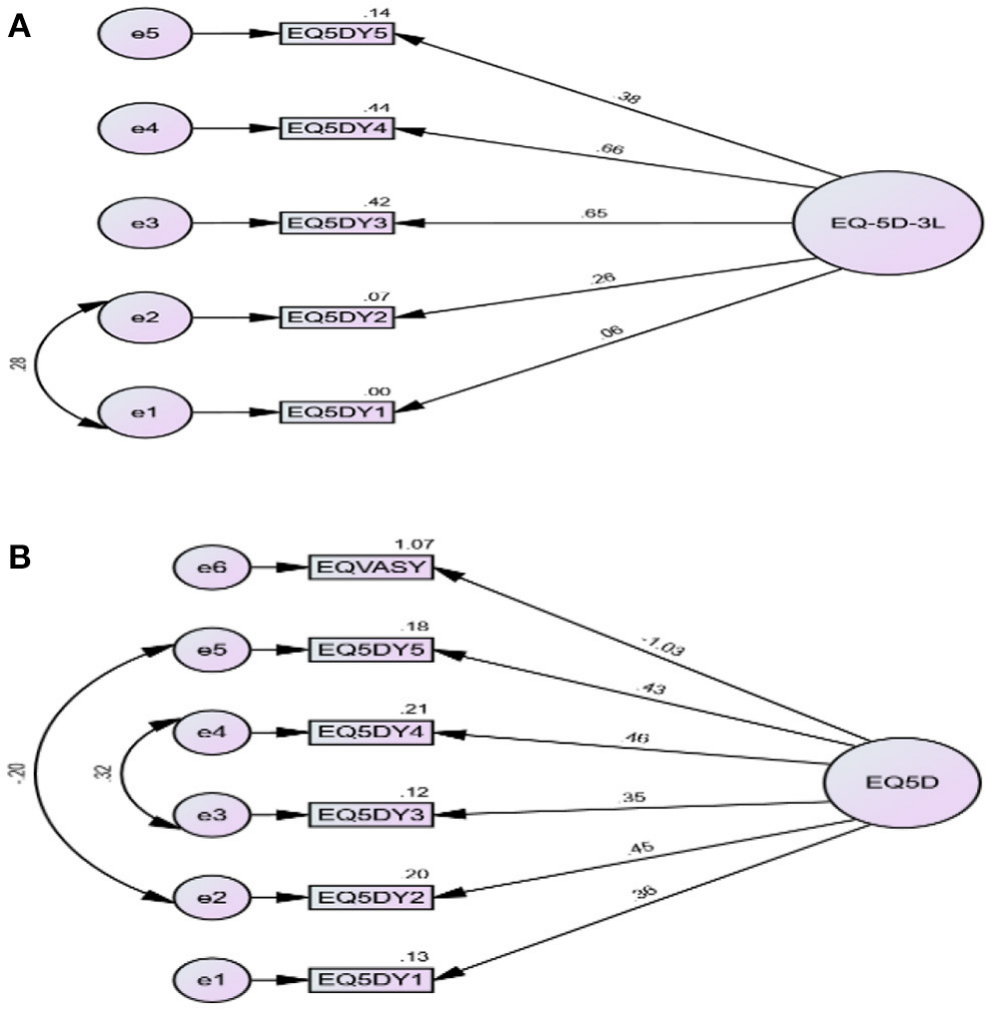
**(A,B)** Structural equation modeling of EQ-5D**-**Yor.

The 1-factor model of EQ-5D returned close fit after modification (maximum of one error modification) (CFI = 1.00, TLI = 1.05, RMSEA = 0.00) (Table 4; Figure 2A). However, when EQ-5D (VAS) was included in the model, it returned satisfactory model fit after modification (maximum of 2-error modification) (CFI = 0.97, TLI = 0.93, RMSEA = 0.08) (Table 4; Figure 2B). The factor loadings were satisfactory in two items of the EQ-5D model (items 3 and 4). However, the factor loadings were poor when EQ-5D (VAS) was included in the model. The composite reliability was satisfactory for the model when VAS was included (0.70) (Table 4).

**Table 4:** Confirmatory factor analysis of the EQ-5D-Y questionnaire.

**Item**	**EQ-5D Model**			**EQ-5D + EQ-VAS Model**		
	**Factor loading**	***R*** ^**2**^	**Composite reliability**	**Factor loading**	***R*** ^**2**^	**Composite reliability**
1	0.064	0.004	0.509	0.361	0.130	0.699
2	0.262	0.069		0.446	0.199	
3	0.651	0.423		0.349	0.122	
4	0.662	0.439		0.460	0.211	
5	0.379	0.144		0.429	0.184	
EQ-5D-Yor (VAS)				−1.033	1.068	
EQ-5D-Yor (Sum)						
Fit indices	*X*^2^ = 3.138, *p* = 0.535, CFI = 1.00, TLI = 1.05, RMSEA = 0.00 (0.00–0.13)			*X*^2^ = 11.371, *p* = 0.123, CFI = 0.97, TLI = 0.93, RMSEA = 0.08 (0.00–0.15)		

*EQ-5D-Yor (VAS), Yoruba translated EuroQol Visual Analog Scale; EQ-5D-Yor (Sum), Sum of all items of the Yoruba translated EuroQol*.

### Convergent Validity of the EQ-5D-Yor Using the SF-12 (Yoruba Version)

There was weak correlation between each of EQ-5D (SUM) and EQ-5D (VAS) with items of SF-12. Only PF of SF-12 had a strong correlation with EQ-5D (SUM) (*r* = −0.709; *p* = 0.409) but not significant (Table 5).

**Table 5:** Correlation of each of the EQ-5D-Yor (Sum) and EQ-5D-Yor (VAS) with the scales and domains of the Yoruba version of the SF-12 (Convergent validity).

**Items**		**Scales**							**Domains**	
	PF	RLP	BP	HP	EF	SF	RLM	MH	MHD	PHD
	*r* (*p*)	*r* (*p*)	*r* (*p*)	*r* (*p*)	*r* (*p*)	*r* (*p*)	*r* (*p*)	*r* (*p*)	*r* (*p*)	*r* (*p*)
EQ-5D-Y SUM	−0.709 (0.409)	−0.390 (0.683)	−0.176 (0.062)	0.088 (0.354)	0.017 (0.860)	0.111 (0.243)	−0.004 (0.969)	0.141 (0.135)	0.047 (0.623)	0.206 (0.030)
EQ-5D-Y VAS	0.016 (0.864)	0.068 (0.471)	0.071 (0.425)	0.027 (0.773)	0.060 (0.526)	0.002 (0.985)	−0.110 (0.246)	−0.061 (0.518)	0.113 (0.234)	0.090 (0.343)

*Scales: PF, Physical functioning; RLP, role limitations due to physical problems; BP, bodily pain; RLM, role limitations due to mental health; SF, social functioning; MH, mental health*.

*Domains: MHD, Mental health domain; PHD, physical health domain; EQ-5D-Yor (VAS), Yoruba translated EuroQoL Visual Analog Scale; Q-5D-Yor (Sum), Sum of all items of the Yoruba translated EuroQol; SF-12-Y, Yoruba translated SF-12*.

### Divergent Validity of the EQ-5D-Yor Using the Visual Analog Scale (Yoruba Version)

A moderate negative correlation of *r* = −0.482 (*p* = 0.001) was found for the divergent validity of the EQ-5L Yor and the Yoruba version of the VAS.

## Discussion

This is the first study to translate, cross-culturally adapt, and determine the reliability and validity of the Yoruba version of the EQ-5D questionnaire. Also, to our knowledge, this is the second translation of the EQ-5D to any sub-Sahara African language. Therefore, this study meets the imperativeness for an increase in cross-cultural research, and the need to adapt health status measures for use in languages other than the source language (18). This is because language can be a barrier to communication within health care, and as such may lead to misinterpretation of information that could easily have grave consequences. For this reason, the EQ-5D has been adapted into various languages such as Korean (6), Singaporean Malay and Tamil (7), Spanish (8), and Chichewa (9).

From the result of this study, the EQ-5D-Yor had a high rate of data completion, with good data obtained. The high response rate among the respondents in the study suggests that the EQ-5D-Yor was an acceptable tool for measuring health perceptions in the general Yoruba population. Furthermore, the concurrent validity of the EQ-5D-Yor ranged from 0.438 to 1.000. This finding suggests that the EQ-5D-Yor compares well with the original version of the EQ-5D. Thus, the EQ-5D is a valid tool for the measurement of health status among Yoruba-speaking populations.

Convergent validity of the EQ-5D-Yor was determined using the Yoruba version of the SF-12 tool. From the result, the EQ-5D-Yor (Sum) (summation of scores of the five dimensions) showed a greater correlation with the physical functioning scale of the SF-12 than any of its other scales/domains, though not statistically significant. This finding is similar to previous studies by Fang et al. (19), which showed a correlation between the EQ-5D items, and the physical domain of the SF-12 compared with the mental health component of the SF-12 survey. On the other hand, the VAS as a generic tool that is often employed to measure different constructs on a scale of 0–10, was used to test the divergent validity of the EQ-5D-Yor. The divergent validity of the EQ-5D-Yor with VAS yielded a moderate negative but significant correlation. One possible reason for the inverse correlation is that both tools do not measure similar constructs, though pain as a construct, is typically correlated with poor HRQoL.

Other results from this study showed that the EQ-5D-Yor was reliable. The least correlated co-efficient score (*r*) was 0.438 (0.001) which is within the moderate correlation range. Multi-trait scaling assumption was used in checking the hypothesized scale structure of the EQ-5D-Yor. Based on this assumption, ICC results were greater than the minimum value of 0.4 recommended by Ware and Gandek (7), which showed a high level of item internal consistency for all items. As expected, all items correlated better with its hypothesized scale than scales measuring other concepts. The internal consistency using Cronbach's alpha at the level of items, three out of five items had Cronbach's alpha >0.7, which is the standard minimum reliability efficient for group-level analyses (20). The ICC was also acceptable because three of five EQ-5D-Yor items scored >0.7, which is the standard minimum measure (11). The results of this study indicated that the EQ-5D-Yor is a tool that can be used to assess HRQoL among the Yoruba-speaking population. This study also revealed that the EQ-5D-Yor has reasonable psychometric properties that are satisfactory within recommended benchmarks.

This study has a few limitations. Firstly, the study population was not homogeneous to patients with any particular musculoskeletal disorder. Thus, there may be an overrepresentation of patients with a particular musculoskeletal disorder. Secondly, pain intensity was not considered in recruiting patients into the study. Thus, individuals with greater or lesser pain intensity may be overrepresented, as pain is often associated with health-related quality of life. Lastly, it is an anecdotal observation in the study setting that patients with chronic illnesses often report or speak in line with their anticipation for better health outcomes rather than report exactly their present health status. This practice is more apparent when the questions in the assessment tool tend toward negative connotations. Therefore, this practice makes the assessment of psychosocial construct in this setting still subjective and with personal idiosyncrasies of the respondents that are often fueled by religion and cultural leanings.

## Conclusion

The EQ-5D-Yor has acceptable validity and reliability and can be used as a valid tool to assess HRQoL in sub-Saharan Africa among the Yoruba-speaking population. The use of country-specific EQ-5D values may have a major impact on QALYs and cost-effective outcomes. Further studies aimed at the translation and adaptation of EQ-5D-5L in different cultural settings in Nigeria could be considered.

## Data Availability Statement

The original contributions presented in the study are included in the article/Supplementary Material, further inquiries can be directed to the corresponding author.

## Ethics Statement

Ethical approval for the study was obtained from the Health Research Ethics Committee, Institute of Public Health, College of Health Sciences, Obafemi Awolowo University, Ile-Ife, Nigeria (IPH/OAU/12/1803). The patients/participants provided their written informed consent to participate in this study.

## Author Contributions

FF, CF, and CM contributed to conception and design of the work. AA, TO, and CM participated in data acquisition. CM, TG, and OO contributed to data analysis. All authors contributed to the manuscript draft and approved the submission.

## Conflict of Interest

The authors declare that the research was conducted in the absence of any commercial or financial relationships that could be construed as a potential conflict of interest.

## Publisher's Note

All claims expressed in this article are solely those of the authors and do not necessarily represent those of their affiliated organizations, or those of the publisher, the editors and the reviewers. Any product that may be evaluated in this article, or claim that may be made by its manufacturer, is not guaranteed or endorsed by the publisher.
