# Supplementary material for: Translation, Cross-Cultural Adaptation, and Psychometric Testing of Yoruba Version of the EQ-5D Questionnaire in Patients With Musculoskeletal Disorders
Source: Front Public Health. 2022 Jun 27;10:902680. doi: 10.3389/fpubh.2022.902680 (PMC9271675; doi:10.3389/fpubh.2022.902680)
Supplement: Supplementary file 1 [file Data_Sheet_1.pdf]

Ìbéèrè lórí Ìlera EQ – 5D   Asamulo titun ☐   **Asamulo atijo** ☐

Àmì Ìdánimo. ☐

Déètì ☐

Jowo´ to´ka sí gbólóhùn tí ó se àpèjúwe tó dára jùlo nípa bí ètò ìlera se rí báyìí nípa fífi àmì sí sokan nínú àwon gbólóhùn ó wà ní owkoo kan nísàle..

Ririn soke sodo

Mi ò ní ìsòro kankan tí mo bá ń rìn kiriMo ní ☐

ìsòro bí mo bá ń rìn kiri ☐

Mo wà ni ihamo ibùsùn mi ☐

Ìtojú ara eni

Mi ò ní ìsòro pelu sise itojú ara mi ☐

Mo ní awon ìsòro díe .bí mo bá ń we tàbí múra. ☐

Mi ò lè dá we .tàbí múra fún ara mi ☐

Àwọn ìse gbogbo ìgbà (bi apeere, ise síse, ìwé kíkà, ise ilé, fàájì tó ní íse pelú ebí)

Mi ò ní ìsòro kankan pelu sise awon akitiyan igbakuugba ☐

Mo ní àwon ìsòro pelu sise akitiyan igbakuugba mi ☐

Mi ò le se awon akitiyan igbakuugba mí ☐

Ìrora/Ìnira

Mi ò ní ìrora/ìnira ☐

Mo máa ń ní ìrora/ìnira níwon. ba Mo ☐

máa ń ní ìrora/ìnira gan ☐

Àniyan/Ìrewesì Okan

Mi ò saniyan/ni ìrewesì okan

Mo ń se aniyan/ní ìrewesì okàn niwonba

Mo n se aniyanè/ní ìrewesì gan

Jowo′ to′k a sí bí ìlera re se dára
tàbí burú sí lónìí lórí síléèlì yìí.

Ìlera tí ó dára jù tí o lè fi ojú inú wò ni
100 (ogorùnún), nígbà tí èyí tí ó burú jù (òdo).

Ipò
ìlera re
lónìí

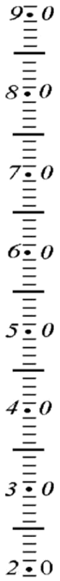

Jowo′ . fa ìlà sí ogangan ibi tí ó so bí ìlera re se dára
tàbí burú sí lórí síkéèlì yìi

ìlera tó burú jái
tí a lè fi ojú inú wò
